# Ginkgolide B Reduces Atherogenesis and Vascular Inflammation in ApoE^−/−^ Mice

**DOI:** 10.1371/journal.pone.0036237

**Published:** 2012-05-11

**Authors:** Xiyun Liu, Gexin Zhao, Yan Yan, Li Bao, Beidong Chen, Ruomei Qi

**Affiliations:** Beijing Institute of Geriatrics, Beijing Hospital and Key Laboratory of Geriatrics, Ministry of Health, Beijing, China; Virginia Commonwealth University Medical Center, United States of America

## Abstract

**Aims:**

To investigate whether ginkgolide B (a platelet-activating factor inhibitor) affects vascular inflammation in atherosclerosis-prone apolipoprotein E-deficient (ApoE^−/−^) mice.

**Methods and Results:**

Human platelets were used to evaluate the effects of ginkgolide B on platelet aggregation and signal transduction. Ginkgolide B attenuated platelet aggregation and inhibited phosphatidylinositol 3 kinase (PI3K) activation and Akt phosphorylation in thrombin- and collagen-activated platelets. ApoE^−/−^ mice were administered a high-cholesterol diet for 8 weeks. Plasma platelet factor 4 (PF4) and RANTES (regulated upon activation, normal T-cell expressed, and secreted protein) were then measured using an enzyme-linked immunosorbent assay. Scanning electron microscopy and immunohistochemistry were used to determine atherosclerotic lesions. Ginkgolide B decreased plasma PF4 and RANTES levels in ApoE^−/−^ mice. Scanning electron microscopic examination showed that ginkgolide B reduced aortic plaque in ApoE^−/−^ mice. Immunohistochemistry analysis demonstrated that ginkgolide B diminished P-selectin, PF4, RANTES, and CD40L expression in aortic plaque in ApoE^−/−^ mice. Moreover, ginkgolide B suppressed macrophage and vascular cell adhesion protein 1 (VCAM-1) expression in aorta lesions in ApoE^−/−^ mice. Similar effects were observed in aspirin-treated ApoE^−/−^ mice.

**Conclusion:**

Ginkgolide B significantly reduced atherosclerotic lesions and P-selectin, PF4, RANTES, and CD40L expression in aortic plaque in ApoE−/− mice. The efficacy of ginkgolide B was similar to aspirin. These results provide direct evidence that ginkgolide B inhibits atherosclerosis, which may be associated with inhibition of the PI3K/Akt pathway in activated platelets.

## Introduction

Growing evidence has shown that platelets are involved in the development of atherosclerosis. However, the contribution of platelets to the process of atherosclerosis is not fully understood [1]–[3]. Platelets are derived from megacaryocytes that possess corpuscle-inflammatory properties. Platelets contain abundant α-granules, dense-granules, and lysosomes where multiple bioactive mediators are stored. Once platelets are activated, these bioactive mediators are released into circulating blood and involved in inflammatory responses.

Platelet factor 4 (PF4; also called CXCL4) belongs to the chemokine family and is stored in platelet α-granules. PF4 accounts for approximately 25% of the proteins in platelet α-granules [4]. PF4 enhances the degranulation of neutrophils primed by tumor necrosis factor (TNF) and promotes their adhesion to endothelial cells. It also enhances the binding of oxidized low-density lipoprotein (LDL) to the LDL receptor on macrophages, human umbilical vein endothelial cells, and vascular smooth muscle cells [5]–[7].

RANTES (regulated upon activation, normal T-cell expressed, and secreted, CCL5) is another inflammatory mediator stored in platelet α-granules and a soluble 7.8 kDa chemokine. Activated platelets can deposit RANTES on the surface of monocytes or atherosclerotic endothelial cells in a P-selectin-dependent process [8], [9]. A recent study reported that blockade of the RANTES receptor attenuates neointima formation and macrophage infiltration in apolipoprotein E-deficient (ApoE^−/−^) mice [10].

Ginkgolide B, an herbal extract from the leaves of the *Ginkgo biloba* tree, is a natural inhibitor of platelet-activating factor (PAF). Previous studies have indicated that ginkgolide B can suppress PAF-mediated platelet activation by competitively binding to the PAF receptor [11], [12]. Our previous studies found that ginkgolide B can suppress oxidized LDL-induced inflammatory protein expression and inhibit nuclear factor-κB (NF-κB) activation in human endothelial cells [13], [14]. However, still unclear is whether ginkgolide B can reduce inflammatory mediators released by platelets in atherosclerosis. The aim of the present study was to evaluate the effects of ginkgolide B on vascular inflammation and atherosclerotic plaque in atherosclerosis-prone ApoE^−/−^ mice.

## Materials and Methods

### Ethics statement

All of the animal experiments were approved by the Institutional Animal Care and Application Committee of the Beijing Institute of Geriatrics (approval no. 20081018), and the investigation conformed with the Guide for the Care and Use of Laboratory Animals published by the United States National Institutes of Health. For the cellular experiments, blood was collected from healthy donors, from whom we received informed consent. The experiment was approved by the Ethics Committee of the Beijing Institute of Geriatrics (approval no. 20081019).

**Figure 1:**
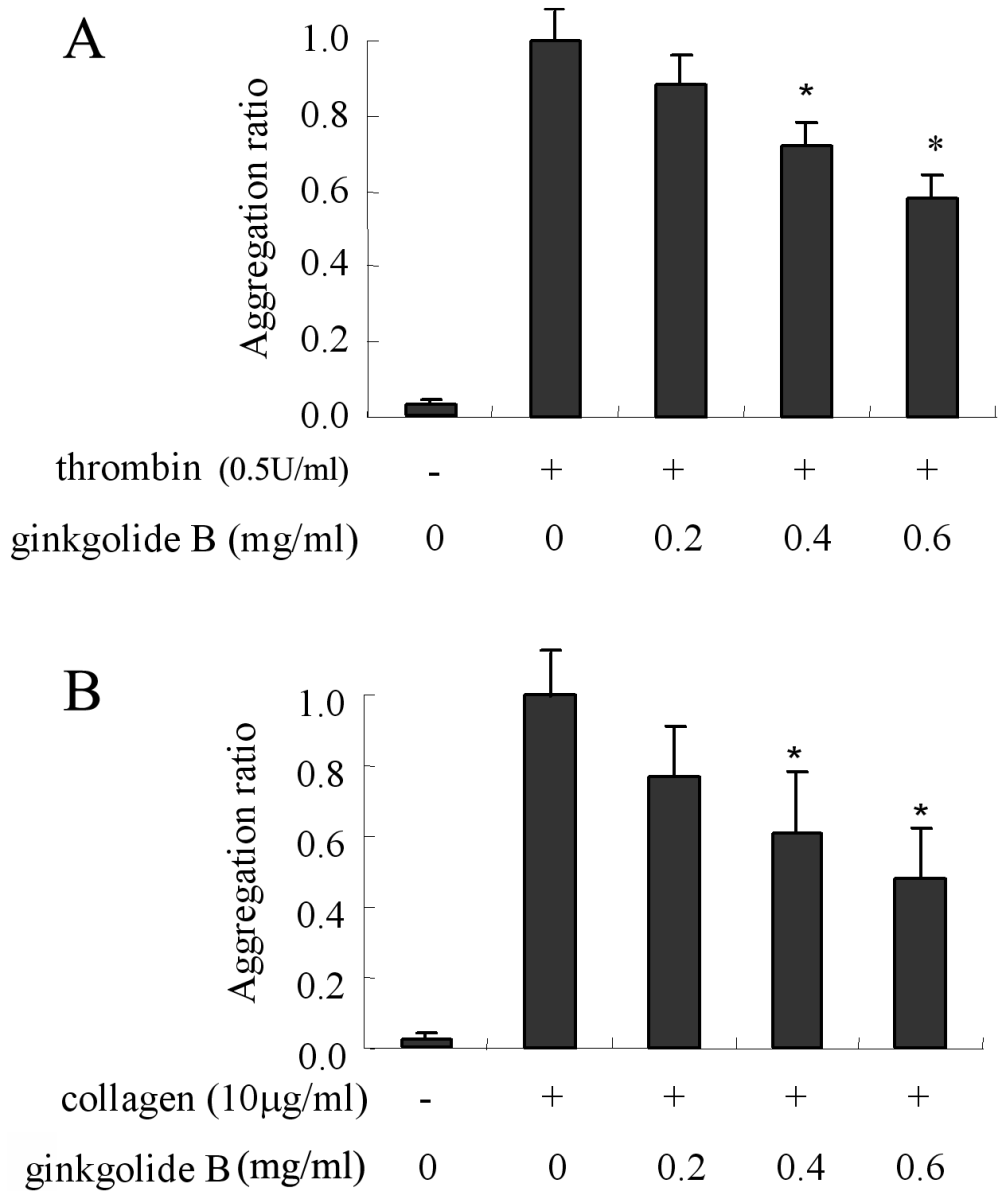
Effect of ginkgolide B on platelet aggregation induced by thrombin and collagen. Various concentrations of ginkgolide B were preincubated with platelets at 37°C for 5 min. Platelet aggregation was then induced by thrombin (0.5 U/ml) or collagen (10 µg/ml). (A) Thrombin-induced platelet aggregation. (B) Collagen-induced platelet aggregation. The data were obtained from five experiments.

**Figure 2:**
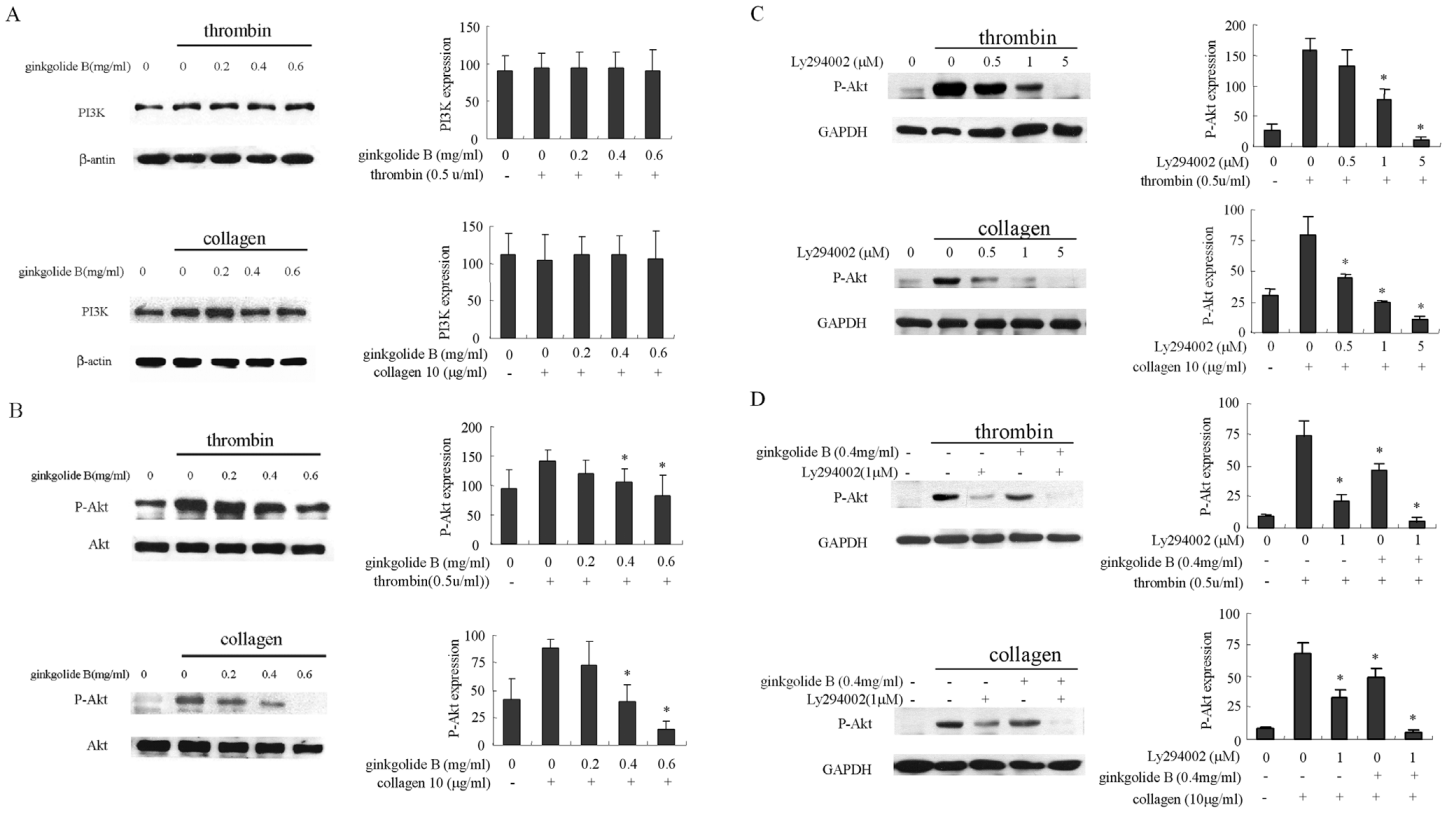
Effect of ginkgolide B on PI3K expression and Akt phosphorylation in activated platelets. Washed platelets were pretreated with various concentrations of ginkgolide B or LY294002 for 5 min. Platelet activation was then challenged by thrombin (0.5 U/ml) and collagen (10 µg/ml). Western blot analysis of PI3K expression and Akt phosphorylation in thrombin- and collagen-activated platelets is shown. (A) Ginkgolide B had no significant effect on PI3K expression in activated platelets. (B) Ginkgolide B blocked Akt phosphorylation in activated platelets. (C) Ly294002 inhibited Akt phosphorylation in activated platelets. (D) The combination of low concentrations of ginkgolide B and Ly294002 completely inhibited Akt phosphorylation. The results were obtained from three independent experiments.

### Materials

Ginkgolide B was purchased from Daguanyuan Company (Xuzhou, China) and had a purity of 95%. PF4 and RANTES enzyme-linked immunosorbent assay (ELISA) kits were purchased from R&D Systems (Minneapolis, MN, USA). Anti-PI3K and anti-Akt antibodies were purchased from Cell Signaling Technologies (Danvers, MA, USA). Anti-P-selectin, anti-RANTES, and anti-PF4 antibodies were purchased from Santa Cruz Biotechnology (Santa Cruz, CA, USA). LY294002 was purchased from Sigma-Aldrich (St. Louis, MO, USA). CD40L antibodies were purchased from Abcam (Boston, MA, USA).

**Figure 3:**
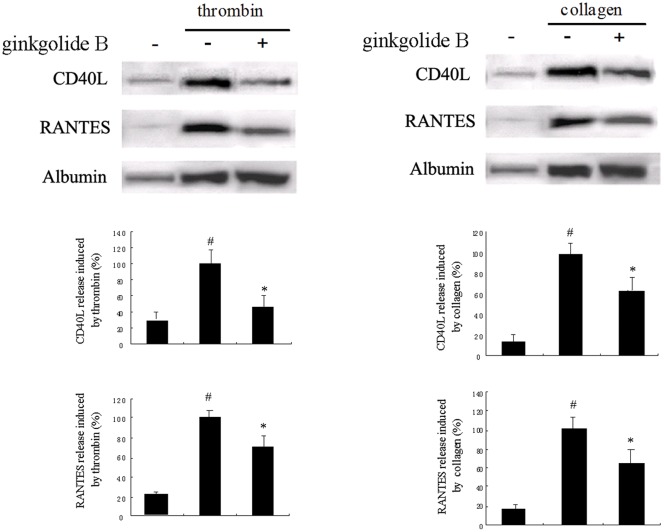
Effect of ginkgolide B on CD40L and RANTES levels in platelets activated by thrombin and collagen. The levels of CD40L and RANTES were determined by Western blot. The results were obtained from three independent experiments. ^#^ *p*<0.05, *vs.* without treatment of platelets; *p<0.05, *vs.* treatment with thrombin alone.

### Plasma PF4, RANTES and Plasma Lipid Measurement

Blood samples were taken by cardiac aspiration after the mice were anesthetized with 1.5% isoflurane. Blood was collected in ethylenediaminetetraacetic acid-coated tubes and centrifuged at 2500× *g* for 15 min at 4°C. Plasma samples were stored at −20°C. Plasma PF4 and RANTES content was assayed using ELISA kits according to the manufacturer’s instructions. Plasma lipid levels were measured by enzymatic method using AU5400 Chemistry System from Beckman Coulter (Kraemer Boulevard Brea, CA, USA).

**Figure 4:**
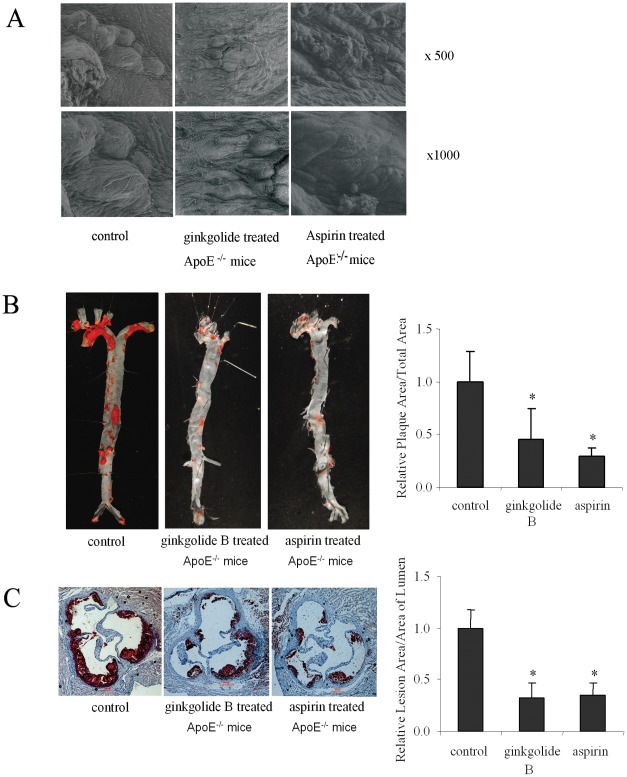
Effect of ginkgolide B and aspirin on atherosclerotic lesion. The mice were sacrificed after being fed high-fat chow for 8 weeks. (A) Scanning electron microscope image of atherosclerotic lesion in each group (*n* = 3 per group). (B) Representative *en face* Oil Red O staining of aorta and quantitative analysis of whole aorta lesion in each group. The percentage lesion area was quantitatively analyzed in each group (*n* = 3 per group). (C) Representative Oil Red O-stained cross-sections from the aortic root in each group. The sections were chosen from mice whose average lesion area approximated the mean value for that group, and the sections shown were obtained at approximately the same level of the aortic root (original magnification, 40×). The percentage lesion area was quantified in each group (*n* = 10 per group).

### Platelet Aggregation Analysis

Citrate anti-coagulated venous blood was obtained from human donors who had not taken any medication for a minimum of 2 weeks before collection. The blood was centrifuged at 400× *g* for 15 min to obtain platelet-rich plasma. The platelets were washed twice in Tyrode/HEPES buffer with 2 mM ethylene glycol tetraacetic acid (EGDA) and resuspended in Tyrode/HEPES buffer at a concentration of 1×10^8^ cells/ml. Platelet aggregation was assessed using a CHRONO-LOG aggregometer.

**Table 1:** Effects of ginkgolide B or aspirin on plasma lipid levels in ApoE^−/−^ mice.

Group	n	TC	TG	HDL-C	LDL-C
control	10	11.42±2.87	0.71±0.30	0.53±0.11	3.43±1.13
ginkgolide B	10	9.87±1.49*	0.55±0.17*	0.58±0.12	2.75±0.63*
aspirin	10	13.02±1.89	0.73±0.18	0.56±0.18	3.92±0.80

Values are in mmol/L (mean±SEM). There were no differences between ginkgolide B group and control or aspirin group and control. *P<0.05, ginkgolide B group *vs* aspirin group.

### Western Blot

Cell lysates were analyzed with sodium dodecyl sulfate-polyacrylamide gel electrophoresis and electrotransferred to polyvinylidene fluoride membranes. The membranes were blocked with 1% bovine serum albumin and then incubated with specific antibodies. After three washes in Tris phosphate-buffered saline (TPBS) that contained 0.5% Tween 20 in PBS, the membranes were incubated with horseradish peroxidase-conjugated secondary antibodies in TPBS. The bands were detected by chemiluminescent detection reagents. Blot densitometry was then performed, and the bands were analyzed using a Gene Genius Bio Imaging System.

**Figure 5:**
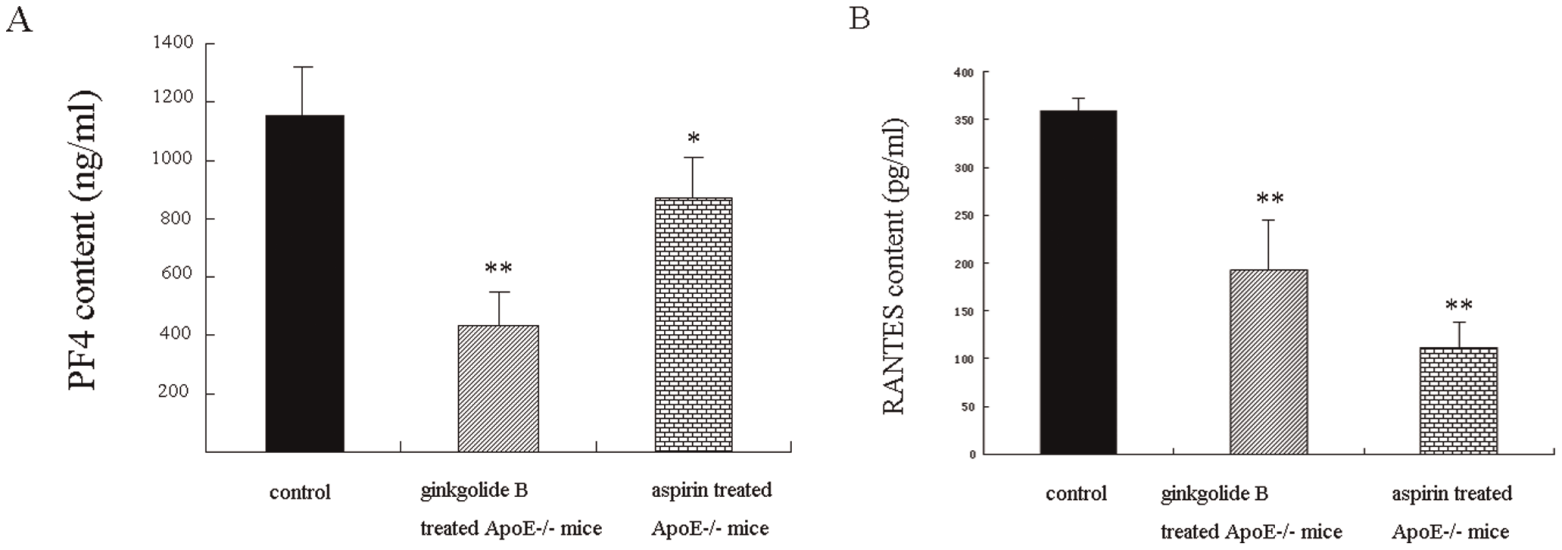
Plasma PF4 and RANTES content was detected by ELISA in each group. All of the mice were administered a high-cholesterol diet for 8 weeks. The data are expressed as mean ± SEM. **p<0.001, *vs.* ApoE^−/−^ mice; *p<0.05, *vs.* control group.

**Figure 6:**
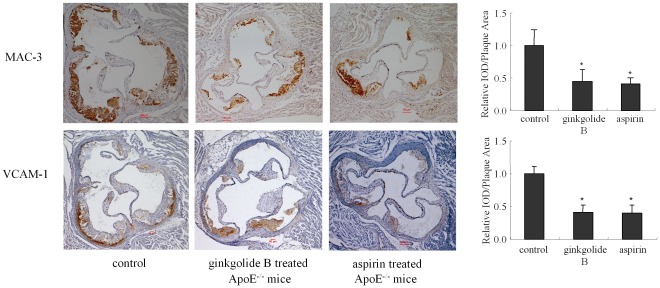
Effect of ginkgolide B and aspirin on macrophage and VCAM-1 expression in plaque in Apo^−/−^ mice. (A) Macrophage expression in plaque in ApoE^−/−^ mice. (B) VACM-1 expression in ApoE^−/−^ mice. The cross-sections were from the aortic root in each group, and these were chosen from mice whose average lesion area approximated the mean value for that group. The sections shown were obtained at approximately the same level of the aortic root (original magnification, 40×). The percentage lesion area was quantified in each group (*n* = 6 per group). *p<0.05, *vs.* control group.

### Animal Experiments

Eight-week-old male ApoE^−/−^ mice (C57/B16 genetic background) were obtained from the Experimental Animal Laboratory of Beijing University (Beijing, China). ApoE^−/−^ mice were fed a high-fat diet that contained 20% fat and 1.25% cholesterol. The mice were randomly divided into three groups (*n* = 12 per group). The mice were given vehicle (0.3 ml PBS/day; control group) or ginkgolide B (0.6 mg/day) or aspirin (0.5 mg/day) by intragastric administration for 8 weeks. The mice were housed in a room with a 12 h/12 h light/dark cycle and allowed free access to food and water. At the end of the experiments, the animals were sacrificed, and blood samples were collected and analyzed.

**Figure 7:**
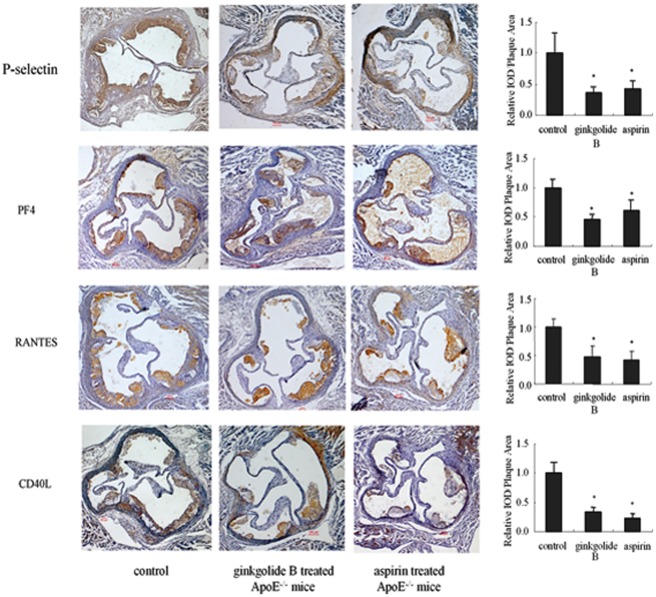
Immunohistochemical analysis of P-selectin, PF4, RANTES, and CD40 expression in plaque in ApoE^−/−^ mice. The cross-sections were from the aortic root in each group, and these were chosen from mice whose average lesion area approximated the mean value for that group. The sections shown were obtained at approximately the same level of the aortic root (original magnification, 40×). The percentage lesion area was quantified in each group (*n* = 6 per group). *p<0.05, *vs.* control group.

### Imaging of Aorta Surface Characteristics in Mice Using an Environmental Scanning Electron Microscope

The mice were sacrificed after being fed high-cholesterol chow for 8 weeks. The aortic tree was perfused for 10 min with PBS. Following the removal of the surrounding adventitial tissue, the aorta was opened longitudinally from the aortic root and fixed with 2.5% glutaraldehyde overnight. The aorta was then washed three times with PBS. The aorta was fixed with OsO_4_ for 4 h, washed three times with PBS, and then dehydrated using various concentrations of ethanol. Finally, the aorta was adhered to carbon-conductive adhesive on the tray. The characteristics of the aorta surface were imaged using an environmental scanning electron microscope (FEI QUANTA 200).

**Figure 8:**
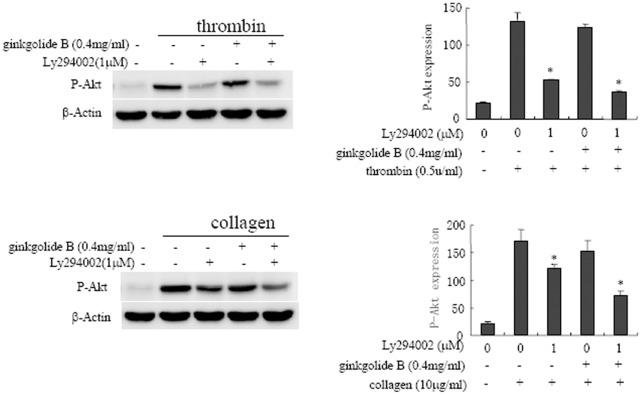
Effect of ginkgolide B on Akt phosphorylation in activated platelets in ApoE^−/−^ mice. Washed platelets in ApoE^−/−^ mice were pretreated with 1 µM LY294002 or 0.4 mg/ml ginkgolide B for 5 min, and platelet activation was then challenged by 0.5 U/ml thrombin or 10 µg/ml collagen. The results were obtained from three independent experiments.

### Atherosclerotic Lesion Size Determination

The proximal part of the thoracic aorta to the aortic origin was isolated from adherent connective tissue and then cleaned. The atherosclerotic plaque area was quantified by analyzing the open luminal surface image of the formalin-fixed aortic arch and thoracic aorta. Atherosclerotic lesions were visible and clearly distinguishable from the non-plaque-covered areas on the luminal surface of the vessels without staining. The colored images were acquired with a Sony digital camera mounted on a microscope (Olympus BX60). The data analysis was performed using image analysis software (Simple, Compix, Mars, PA, USA), and the same setting was retained for the analysis of each group.

**Figure 9:**
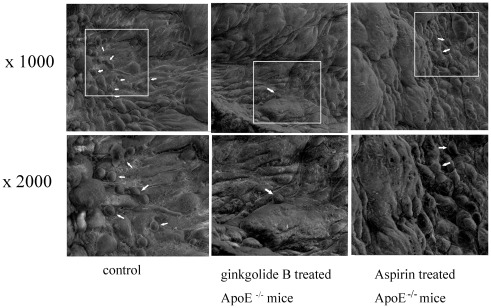
Effect of ginkgolide B on platelet aggregation on atherosclerotic lesion in ApoE^−/−^ mice. The mice were sacrificed after being fed high-fat chow for 8 weeks. Platelet aggregation on atherosclerotic lesions was observed in each group using a scanning electron microscope. Arrows indicate platelet accumulation.

### Immunohistochemistry

The mice were euthanized with 1.5% isoflurane, and the aorta was dissected, opened longitudinally from the heart to iliac arteries, and stained with Oil Red O to determine the lesion area. Specimens that contained the aortic root, aortic arch, thoracic aorta, and abdominal aorta were immersed for 3 s in 60% isopropanol and stained for 10 min in a saturated Oil Red O solution. The specimens were then rinsed in 60% isopropanol for 1 min and washed three times. The aortic root specimens were dissected under a microscope, fixed in a 4% formaldehyde solution, and frozen in an optimal-cutting-temperature embedding medium for serial cryosectioning that covered 0.8 mm of the root. The cryosections (7 µm) were examined with Oil Red O staining and immunohistochemistry. The average immunohistochemical staining with various antibodies (i.e., PF4, RANTES, CD40L, MAC-3, P-selectin, and vascular cell adhesion protein 1 [VCAM-1]) was assessed from multiple samples of each artery. Bound antibody was detected with a DAB substrate kit (Beijing Zhongshan Golden Bridge Biotechnology, Beijing, China). Semiquantitative immunostaining analysis was performed using an Olympus microscope linked to the ImagePro Plus image analysis system (Media Cybernetics, Bethesda, MD, USA).

### Statistical Analysis

The data are expressed as mean ± SEM. Statistical analyses were performed using independent *t*-tests and analysis of variance (ANOVA) followed by the Tukey *post hoc* test. The results were considered significant at values of *p*<0.05.

## Results

### Ginkgolide B Inhibits Platelet Aggregation

In the present study, we first examined whether ginkgolide B inhibits platelet aggregation induced by thrombin and collagen. As shown in Fig. 1, platelets pretreated with ginkgolide B (0.2, 0.4, and 0.6 mg/ml) for 5 min exhibited impaired aggregation ability. Ginkgolide B (0.6 mg/ml) significantly inhibited thrombin- and collagen-induced platelet aggregation. The inhibitory ratio was 43.2% for thrombin-induced platelet aggregation and 51.3% for collagen-induced platelet aggregation.

### Effects of Ginkgolide B on PI3K and Akt Phosphorylation

The PI3K signaling pathway includes a group of enzymes that generate lipid second messengers to mediate multi-signal transduction. The PI3K pathway is involved in thrombin- and collagen-induced platelet activation. To investigate the effects of ginkgolide B on human platelet activation, we first determined PI3K 110β expression and Akt phosphorylation. As shown in Fig. 2A, PI3K expression was slightly increased in stimulated platelets by 0.5 U/ml thrombin and 10 µg/ml collagen, but ginkgolide B had no significant effect on PI3K expression. Akt phosphorylation was then examined. As shown in Fig. 2B, both thrombin and collagen challenged Akt phosphorylation in activated platelets. Ginkgolide B significantly and dose-dependently decreased Akt phosphorylation in thrombin- and collagen-activated platelets. We then investigated the effect of LY294002, a specific PI3K inhibitor, on Akt phosphorylation in activated platelets. As shown in Fig. 2C, LY294002 dose-dependently inhibited Akt phosphorylation in thrombin- and collagen-activated platelets. Furthermore, we used low concentrations of LY294002 and ginkgolide B that failed to fully attenuate Akt phosphorylation alone. As shown in Fig. 2D, the combination of low concentrations of LY294002 and ginkgolide B completely abolished Akt phosphorylation, suggesting that inhibition of Akt phosphorylation by ginkgolide B might occur via inhibition of PI3K activation. We then determined whether ginkgolide B can inhibit platelet release in activated platelets.

### Analysis of Platelet Release

We then determined the effects of ginkgolide B on platelet release induced by thrombin and collagen. The supernatant was collected after platelets were stimulated by thrombin or collagen as described in the Methods. As shown in Fig. 3, treatment with 0.6 mg/ml ginkgolide B potently attenuated CD40L and RANTES release in platelets activated by thrombin and collagen. In the experiment, albumin was used as a reference object for the platelet release response.

### Ginkgolide B Inhibits Aortic Atherosclerotic Lesion Area

To evaluate whether ginkgolide B influences atherosclerosis, we first observed the characteristics of the aorta surface in ApoE^−/−^ mice with 8 weeks of treatment using a scanning electron microscope. As expected, lesion development was seen at the end of the 8-week high-cholesterol diet. As shown in Fig. 4A, a large number of plaques appeared on the surface of the aorta in ApoE^−/−^ mice. In contrast, aortic plaques were reduced in both ginkgolide B- and aspirin-treated ApoE^−/−^ mice. Moreover, we used Oil Red O staining of the aorta in each group. As shown in Fig. 4B, the plaque area decreased by 50.6% in the ginkgolide B-treated group and 63.2% in the aspirin-treated group compared with the control group. Oil Red O-stained serial cross-sections of the aorta showed that the plaque area of the aortic root decreased by 67.2% in the ginkgolide B-treated group and 65.5% in the aspirin-treated group (Fig. 4C). These results provide direct evidence that ginkgolide B and aspirin have protective effects against atherosclerosis in ApoE^−/−^ mice.

### Effects of Ginkgolide B on Plasma Lipid Levels

The mice were fed a high-fat diet for 8 weeks, and plasma lipid levels were analyzed. As shown in Table 1, no significant differences in lipid levels were found between the control group and ginkgolide B-treated group or between the control group and aspirin-treated group. However, significant differences in the levels of TC, TG, and LDL were observed between the ginkgolide B-treated group and aspirin-treated group (*P*<0.05).

### Effects of Ginkgolide B on Plasma PF4 and RANTES

Platelets become activated at the site of vascular injury and secrete their α-granule and dense-granule contents under conditions of atherosclerosis and high lipidemia. Plasma PF4 and RANTES levels were detected by ELISA in the present study. As shown in Fig. 5, ginkgolide B potently reduced the level of PF4 by 62.4% in plasma in ApoE^−/−^ mice compared with the control group. Aspirin also decreased the level of PF4 by 25%. Ginkgolide B abolished the increase in RANTES. Compared with the control group, ginkgolide B reduced RANTES by 46.1%, and aspirin decreased RANTES by 69.2%. These results suggest that the ginkgolide B- and aspirin-induced decreases in the levels of PF4 and RANTES might be associated with platelet function inhibition.

### Effect of Ginkgolide B on Macrophage and VCAM-1 Expression in Plaque

To investigate the effects of ginkgolide B on vascular inflammation in atherosclerosis, immunohistochemical analysis was performed in the aortic root in ApoE^−/−^ mice. The Mac-3 antibody was used for staining macrophages in the aortic root in ApoE^−/−^ mice. We used IPP6.0 software to determine the abundance of macrophages in plaque from three ApoE^−/−^ mice. As shown in Fig. 6A, compared with the control group, ginkgolide B decreased the macrophage distribution by 50.0% in Ape E^−/−^ mice, and aspirin decreased the macrophage distribution by 59.3% in ApoE^−/−^ mice. We then examined VCAM-1 expression in aortic plaque. VCAM-1 expression was reduced by 58.4% in aortic plaque in the ginkgolide B-treated group and 60.4% in the aspirin-treated group (Fig. 6B). These results indicate that ginkgolide B and aspirin suppressed vascular inflammation in ApoE^−/−^ mice.

### Effect of Ginkgolide B on P-selectin, PF4, RANTES, and CD40L in Plaque

We also investigated whether ginkgolide B can abolish P-selectin, PF4, RANTES, and CD40L expression in plaque in ApoE^−/−^ mice. As shown in Fig. 7, both ginkgolide B and aspirin attenuated the plaque expression of these four inflammatory proteins in ApoE^−/−^ mice. Compared with the control group, P-selectin expression in plaque was decreased by 63.4% in the ginkgolide B-treated group and 58.3% in the aspirin-treated group. PF4 expression was decreased by 54.7% in ginkgolide B-treated ApoE^−/−^ mice and 39.2% in aspirin-treated ApoE^−/−^ mice. RANTES expression was decreased by 51.8% in the ginkgolide B-treated group and 58.8% in the aspirin-treated group. CD40L expression was decreased by 66.4% in ginkgolide B-treated ApoE^−/−^ mice and 76.9% in aspirin-treated ApoE^−/−^ mice. These results suggest that both ginkgolide B and aspirin can suppress inflammatory protein expression in plaque in ApoE^−/−^ mice, an effect that is likely attributable to the inhibition of platelet function.

### Effect of Ginkgolide B on Akt Phosphorylation in ApoE^−/−^ Mice

We then determined whether ginkgolide B has similar effects on the PI3K/Akt pathway in ApoE**^−/−^** mice. We collected platelets from ApoE**^−/−^** mice to evaluate the effect of ginkgolide B on Akt phosphorylation in activated platelets. As shown in Fig. 8, Akt phosphorylation was induced in platelets activated by thrombin and collagen. Moreover, concentrations of 1 µM LY294002 and 0.4 mg/ml ginkgolide B alone partially inhibited Akt phosphorylation. The combination of low concentrations of LY294002 and ginkgolide B potently abolished Akt phosphorylation. The results suggest that ginkgolide B inhibits platelet activation in ApoE**^−/−^** mice via a similar mechanism.

### Ginkgolide B Inhibits Platelet Aggregation in Atherosclerotic Lesion

Platelet aggregation was observed in atherosclerotic plaque using a scanning electron microscope. As shown in Fig. 9, many platelets aggregated on the atherosclerotic lesion in ApoE^−/−^ mice. In contrast, few platelets accumulated on plaques in ginkgolide B- and aspirin- treated ApoE^−/−^ mice. The image provides a visual representation of the platelets involved in atherosclerosis.

## Discussion

The present study investigated whether ginkgolide B can decrease the development of atherosclerosis. Aspirin is widely used for its anti-platelet function and is an irreversible cyclooxygenase-1 inhibitor. Recent studies have indicated that aspirin can reduce atherosclerotic lesions and serum TXA2 levels in ApoE^−/−^ mice [15], [16]. Therefore, the effect of ginkgolide B was compared with aspirin on atherosclerosis in ApoE^−/−^ mice. The scanning electron microscope and Oil Red O staining results showed that both ginkgolide B and aspirin significantly reduced atheromatous plaque in ApoE^−/−^ mice. Macrophages and the inflammatory protein VCAM-1 were also proportionally decreased in atheromatous plaque in ApoE^−/−^ mice. Furthermore, the results showed that ginkgolide B and aspirin decreased PF4 and RANTES levels in plasma. Immunohistochemistry showed that both ginkgolide B and aspirin significantly reduced these inflammatory proteins in aortic plaque in ApoE^−/−^ mice. Ginkgolide B appeared to have better inhibitory effects on PF4 expression in plaque, and aspirin had stronger effects on RANTES expression in plaque. These results suggest that the mechanism of platelet function inhibition by ginkgolide B is distinct from the mechanism of aspirin *in vivo*.

The effects of ginkgolide B on the inhibition of platelet function were investigated in the present study. Ginkgolide B significantly inhibited platelet aggregation induced by thrombin and collagen. The PI3K pathway has been shown to play an important role in collagen- and thrombin-induced platelet activation [17], [18]. Previous studies showed that thrombin stimulates platelet α-granule release via the PI3K pathway [19], [20]. Recent studies provided evidence of the crucial role of the PI3K family in the regulation of inflammation within the vasculature during atherogenesis, such as inflammatory cell recruitment and the expression and activation of inflammatory mediators. The results showed that ginkgolide B abolished Akt phosphorylation by inhibiting PI3K activation, whereas ginkgolide B had no effects on PI3K expression. These results may indicate that changes in protein expression may require a longer time to develop in cells. The PI3K family contains various subunits, such as classes IA, IB, II, and III. The precise regulation of PI3K by ginkgolide B needs further study. Additionally, the inhibitory effect of ginkgolide B on Akt phosphorylation was also confirmed in ApoE^−/−^ mice.

Our results also demonstrated that ginkgolide B decreased CD40L and RANTES levels in platelet release products. RANTES is upregulated at sites of vascular inflammation. As a potent chemoattractant, it also mediates monocyte and T-lymphocyte migration through endothelial cell-cell junctions. RANTES receptor antagonists can reduce atherosclerotic plaque formation in mice [21], [22]. CD40 is a transmembrane receptor of the TNF gene superfamily, and more than 95% of the circulating CD40 ligand (CD40L) is derived from platelets. CD40L enhances inflammation by promoting the release of cytokines, chemokines, and cell-cell interactions [23], [24]. A recent study elucidated a novel alternative pathway for CD40L-mediated inflammation that formed interactions with the monocyte/macrophage integrin Mac-1 [25]. PF4 promotes monocyte survival, induces monocyte differentiation into macrophages, and accelerates the formation of foam cells in atherosclerosis [26]. P-selectin is a cell surface adhesion molecule that plays an essential role in the initial recruitment of leukocytes to the site of injury during inflammation [27]. P-selectin has been detected on the surface of endothelia at all stages of atherosclerotic lesion development in humans and animal models of atherosclerosis. Some studies showed that anti-P-selectin antibodies inhibit monocyte rolling on the endothelium of carotid arteries isolated from ApoE^−/−^ mice. The absence of P-selectin delays fatty streak formation in mice [28], [29]. The present results provide direct evidence that ginkgolide B represses atherosclerosis by attenuating P-selectin, RANTES, CD40L, and PF4 expression in plaque in ApoE^−/−^ mice, with efficacy similar to aspirin.

Our data demonstrated that platelets are involved in atherosclerosis, and ginkgolide B inhibited inflammation by attenuating platelet release. The precise mechanism of action and possible clinical applications will need to be addressed in future studies. The effects of ginkgolide B on the stability and accumulation of plaque should also be further evaluated at different time-points in the development of atherosclerosis.

## Conclusions

The present results provide direct evidence that ginkgolide B can attenuate inflammatory protein expression in plaque in ApoE^−/−^ mice. The mechanisms might include the abolition of P-selectin, RANTES, PF4, and CD40L expression. These inflammatory mediators are closely related to platelet release. These results suggest that ginkgolide B can attenuate atherosclerosis in ApoE^−/−^ mice and inhibit platelet release by blocking the PI3k/Akt pathway in thrombin- and collagen-activated platelets. These results also suggest the therapeutic potential of ginkgolide B in the prevention and treatment of atherosclerosis.
